# Adsorption and Permeation Events in Molecular Diffusion

**DOI:** 10.3390/molecules29215012

**Published:** 2024-10-23

**Authors:** Denis S. Grebenkov

**Affiliations:** 1 CNRS – Université de Montréal CRM—CNRS, 6128 Succ Centre-Ville, Montréal, QC H3C 3J7, Canada; denis.grebenkov@polytechnique.edu; 2 Laboratoire de Physique de la Matière Condensée, CNRS—Ecole Polytechnique, Institut Polytechnique de Paris, 91120 Palaiseau, France

**Keywords:** diffusion, surface reaction, permeation, heterogeneous catalysis, confinement, geometric complexity, biochemistry, reversible reactions, encounter-based approach, Brownian motion

## Abstract

How many times can a diffusing molecule permeate across a membrane or be adsorbed on a substrate? We employ an encounter-based approach to find the statistics of adsorption or permeation events for molecular diffusion in a general confining medium. Various features of these statistics are illustrated for two practically relevant cases: a flat boundary and a spherical confinement. Some applications of these fundamental results are discussed.

## 1. Introduction

Many chemical and biological phenomena are governed by molecular diffusion [1,2,3,4,5,6,7], with examples ranging from oxygen capture by blood in the lungs and placenta [8,9,10,11] to proteins searching for enzymes or specific sites on a DNA chain [12,13,14,15]. In such diffusion-controlled reactions, the diffusion-driven mixture of reacting molecules or their diffusive transport to immobile reactive patches are relevant and often limiting factors in the overall chemical kinetics [16,17,18,19,20,21]. In addition, the diffusing molecules can reversibly bind to other molecules or adsorb/desorb on a substrate [22,23,24,25,26,27,28,29]. For instance, reversible binding of calcium ions to buffer molecules controls the cascade of biochemical reactions in the presynaptic bouton and thus signal transduction between neurons [30,31,32,33,34]. Moreover, if the confining medium is compartmentalized by semi-permeable membranes, the molecules can permeate across them and thus explore different compartments [35,36,37,38,39,40,41,42]. While the theory of such reversible reactions is rather well developed (see reviews [21,29] and references therein), the statistics of adsorption and permeation events remain poorly understood. How many times would a single molecule cross the membrane or experience adsorption/desorption up to a given time or before its escape, degradation, passivation, or ultimate transformation? This fundamental question can shed light on the intricate diffusive dynamics of molecules in complex media and help in the design of more efficient mechanisms and protocols for targeted drug delivery [43,44].

Different mathematical tools were employed to study the dynamics of reversible diffusion-controlled reactions. Some theoretical models substitute the spatial dynamics of a molecule by a random walk on a lattice [45,46,47,48,49,50,51], in which case the number of adsorption or permeation events can be easily introduced. However, the obtained statistics depend on the lattice size, and finding its asymptotic behavior presents a difficult task. Moreover, the related analytical computations rely on combinatorial analysis and are generally accessible only for few simple geometric settings. As a consequence, one usually employs another framework, in which the dynamics of a molecule is described as Brownian motion, restricted by reflecting walls of the confinement. In this framework, one can consider either the macroscopic concentration of molecules, or the diffusive propagator characterizing the random position of a single molecule. In both cases, theoretical or numerical analysis focuses on the diffusion equation with appropriate boundary conditions, for which various mathematical tools are available. In particular, when the target is small or the confinement is large, matched asymptotic analysis and scaling arguments yield an accurate description of first-passage times (see [52,53,54,55,56,57,58,59] and references therein). At the same time, the very definition of an adsorption or permeation event is becoming more sophisticated. In fact, when Brownian motion arrives at a reflecting boundary, it exhibits infinitely many reflections within an infinitely short time period [60]. This paradoxical property is a mathematical consequence of the self-similarity of Brownian motion at all scales. Accordingly, if an adsorption or permeation event is initiated upon the first arrival onto the boundary, the molecule cannot leave it to resume its motion in the bulk, as it will be immediately re-adsorbed. To overcome this technical difficulty, one can realize desorption or permeation as a jump from the boundary into the bulk to a small distance a>0 [61,62], which may represent the finite thickness of a reactive surface layer, the thickness of a permeable membrane, or the short range of interactions between the molecule and the boundary. Alternatively, one can introduce finite reactivity, permeability, or binding affinity κ of the molecule to the boundary so the molecule has a finite probability to adsorb or permeate at each arrival onto the boundary [63,64,65,66,67,68,69,70,71,72,73]. Both methods yield similar results when *a* is small, but the second option has the advantage of preserving the continuity of random trajectories.

Recently, a more rigorous formulation of adsorption and permeation events was established [74,75,76,77]. This so-called encounter-based approach relies on the boundary local time $\ell_{t}$, a mathematical process that represents the rescaled number of encounters between the molecule and the boundary up to time *t*. This formulation provides a rigorous foundation for the earlier works based on the finite reactivity κ and allows one to study much more general binding and permeation mechanisms. This approach turns out to be particularly suitable for describing a sequence of diffusive explorations near the boundary after each failed adsorption/permeation attempt. In this paper, we employ the encounter-based approach to obtain the exact statistics of adsorption or permeation events. As permeation across the membrane involves diffusion in distinct compartments, its analysis is much more difficult. To avoid the related technicalities and to keep the presentation to an accessible level, we focus mostly on the adsorption events. After deriving the general solution for the statistics of adsorption events (Section 2) and permeation events (Section 3), we present two relevant examples of adsorptions on a flat boundary and on a spherical boundary (Section 4). In both cases, we manage to obtain rather explicit formulas to illustrate how the statistics of adsorption events depend on other parameters. Section 5 concludes the paper with a summary of the main findings and future perspectives.

## 2. Statistics of Adsorption Events

We consider molecular diffusion in a Euclidean domain $\Omega \subset R^{d}$ whose boundary $\partial \Omega =\Gamma \cup \partial \Omega_{0}$ is split into two disjoint parts: an adsorbing surface Γ and a reflecting inert boundary $\partial \Omega_{0}$. For a molecule starting from a point $x_{0}\in \Omega$, we aim to characterize the statistics of the number $N_{t}$ of adsorptions on Γ up to time *t*. This setting resembles the escape problem for a sticky particle [78,79]. The difference here is that we focus on a fixed time *t*, whereas the escape problem corresponds to $N_{T}$, with T being the first-escape time. For a better understanding of these statistics, we assume that the adsorption events are infinitely short, i.e., an adsorbed molecule is immediately released and resumes its diffusion until the next adsorption. Note that random durations of adsorption events can be easily incorporated into the proposed formalism (see the paragraph after Equation (21)).

First of all, one needs to formulate the adsorption/desorption mechanism. We follow the encounter-based approach, in which the adsorption event occurs when the number of encounters between the molecule and the adsorbing boundary Γ exceeds some threshold [61,80]. In more rigorous terms, the random adsorption time T is defined as $T=\inf\{t>0\;:\;\ell_{t}>\hat{\ell }\}$, where $\ell_{t}$ is the boundary local time on Γ (i.e., a rescaled number of encounters with Γ up to time *t*) and $\hat{\ell }$ is the random threshold with a given distribution [74]. For instance, if $\hat{\ell }$ obeys an exponential law, $P\left\{\hat{\ell }>\ell \right\}=e^{-q\ell }$ with some q>0, this mechanism describes adsorption to the boundary with a constant reactivity κ=qD, governed by Robin boundary conditions [74,77]. In the following, we will treat more general adsorption mechanisms characterized by a given probability law $\Psi \left(\ell \right)=P\left\{\hat{\ell }>\ell \right\}$ for the threshold $\hat{\ell }$, and then discuss the special case of constant reactivity. In this framework, the desorbed molecule can be released from the adsorption point on the boundary Γ, thus preserving the continuity of the trajectory. As each desorption event is associated with resetting of the boundary local time $\ell_{t}$ to zero, the molecule needs to encounter the boundary a number of times before being re-adsorbed, resulting in well-defined adsorption/desorption kinetics. Instead of resetting the boundary local time $\ell_{t}$, one can equivalently introduce a sequence of independent thresholds ${\hat{\ell }}_{1},{\hat{\ell }}_{2},\ldots ,{\hat{\ell }}_{n}$ to define a sequence of adsorption times,

(1) $$T_{n}=\inf\left\{t>0\;:\;\ell_{t}>{\hat{\ell }}_{1}+\ldots +{\hat{\ell }}_{n}\right\}\;\left(n=1,2,\ldots \right).$$

**Figure 1:**
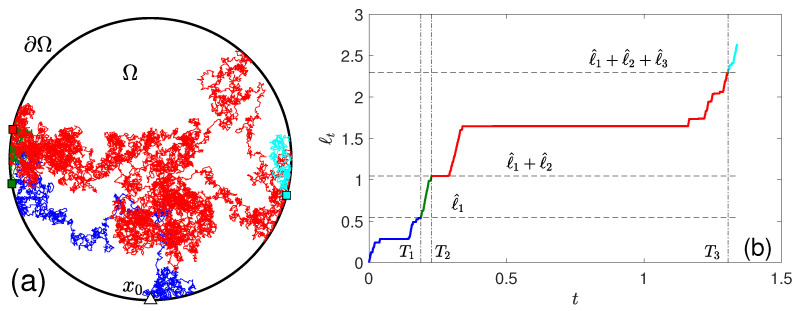
(**a**) A simulated trajectory of a molecule diffusing inside a disk Ω with an adsorbing circular boundary Γ=∂Ω. A triangle indicates the starting point $x_{0}$, while three filled squares show three positions, at which the molecule was adsorbed to the boundary. (**b**) The associated boundary local time $\ell_{t}$ (obtained along the same simulation) crosses three random thresholds ${\hat{\ell }}_{1}$, ${\hat{\ell }}_{1}+{\hat{\ell }}_{2}$, and ${\hat{\ell }}_{1}+{\hat{\ell }}_{2}+{\hat{\ell }}_{3}$ (horizontal lines) at random times $T_{1}$, $T_{2}$, and $T_{3}$ (vertical lines). Each such crossing corresponds to an adsorption on the boundary. Colors distinguish successive time periods between adsorption events.

Figuratively speaking, thresholds ${\hat{\ell }}_{1}$, ${\hat{\ell }}_{1}+{\hat{\ell }}_{2}$, etc., can be interpreted as “milestones” on a road, whereas $T_{1}$, $T_{2}$, etc., are the successive crossing times of these milestones. For instance, Figure 1a illustrates a simulated trajectory of a molecule diffusing inside a disk, with three adsorptions on its boundary (shown by filled squares). The *n*-th adsorption occurs at a random time $T_{n}$ when the boundary local time $\ell_{t}$ exceeds the random threshold ${\hat{\ell }}_{1}+\ldots +{\hat{\ell }}_{n}$. The simulated trajectory of the boundary local time $\ell_{t}$ and the corresponding thresholds are shown in Figure 1b. We recall that $\ell_{t}$ is a non-decreasing stochastic process that increments only at encounters with the boundary (see [74,80] and references therein); in particular, $\ell_{t}$ remains constant when the molecule diffuses in the bulk, and such periods of “stagnation” can be macroscopically long.

We are interested in describing the statistics of the number of adsorptions up to time *t*:

(2) $$N_{t}=\max\left\{n\geq 0\;:\;T_{n}<t\right\}$$

(with $T_{0}=0$), i.e., the number of adsorption times $T_{n}$ that do not exceed *t*. Since $N_{t}$ is a discrete stochastic process, we can fully characterize it by finding the probabilities $Q_{n}\left(t|x_{0}\right)=P\left\{N_{t}=n\right\}$, with n=0,1,2,….

While our main emphasis is on the statistics of $N_{t}$ for a fixed time *t*, another important statistic can be considered. For this extension, we assume that the diffusing molecule has a finite random lifetime τ [81,82,83]. This lifetime can account for eventual degradation or passivation of the molecule, or its irreversible transformation into another (inactive) molecule. What is the distribution of the random variable $N_{\tau }$—the number of adsorption events experienced by a molecule during its lifetime? If the random variable τ is independent of the dynamics, the statistics of $N_{\tau }$ can be obtained by averaging the statistics of $N_{t}$ with the PDF ρ(t) of τ:

(3) $$Q_{n}\left(x_{0}\right)=E\left\{Q_{n}\left(\tau |x_{0}\right)\right\}=\int_{0}^{\infty }dt\;\rho \left(t\right)\;Q_{n}\left(t|x_{0}\right),$$

where E{·} denotes expectation (or ensemble average). In the most common case of an exponentially distributed lifetime, $\rho \left(t\right)=pe^{-pt}$ (with the rate p>0 or mean lifetime 1/p), the right-hand side of this expression is proportional to the Laplace transform of $Q_{n}\left(t|x_{0}\right)$:

(4) $$Q_{n}\left(x_{0}\right)=p\int_{0}^{\infty }dt\;e^{-pt}\;Q_{n}\left(t|x_{0}\right)=p\;{\tilde{Q}}_{n}\left(p|x_{0}\right),$$

where tilde denotes the Laplace transform with respect to time. In the next subsection, we will derive a rather explicit form for the Laplace transform ${\tilde{Q}}_{n}\left(p|x_{0}\right)$ so that the statistics of such $N_{\tau }$ is actually much simpler than that of $N_{t}$.

### 2.1. Encounter-Based Approach

In order to compute $Q_{n}\left(t|x_{0}\right)$, we employ the encounter-based approach [74]. Following this reference, we introduce the generalized propagator $G(x,t|x_{0})$, i.e., the probability density for a molecule started from $x_{0}\in \Omega$ at time 0 to be found in a vicinity of x∈Ω at time *t* without being adsorbed up to *t*. Since the no adsorption condition corresponds to $\ell_{t}>\hat{\ell }$, the generalized propagator can be written as

(5) $$G\left(x,t|x_{0}\right)=\int_{0}^{\infty }d\ell \;\Psi \left(\ell \right)\;P\left(x,\ell ,t|x_{0}\right),$$

where $P(x,\ell ,t|x_{0})$ is the encounter propagator, i.e., the joint probability density of finding the molecule at point x with the boundary local time *ℓ* at time *t*, given that it started from $x_{0}$. The generalized propagator determines the probability flux density, $j\left(x,t|x_{0}\right)=-D\partial_{n}G\left(x,t|x_{0}\right){|}_{\Gamma }$, where $\partial_{n}$ is the normal derivative oriented outwards the domain Ω. This is the joint probability density of the adsorption time T and the position $X_{T}$ of the adsorption event on the boundary Γ. The Markovian property of the diffusive dynamics allows us to write

(6) $$\begin{array}{cc} Q_{0}\left(t|x_{0}\right) & =S(t|x_{0}),\\ Q_{n}\left(t|x_{0}\right) & =\int_{\Gamma }dx_{1}\int_{0}^{t}dt_{1}\;j\left(x_{1},t_{1}|x_{0}\right)\int_{\Gamma }dx_{2}\int_{t_{1}}^{t}dt_{2}\;j\left(x_{2},t_{2}-t_{1}|x_{1}\right)\ldots \end{array}$$

(7) $$\begin{array}{cc} \; & \times \int_{\Gamma }dx_{n}\int_{t_{n-1}}^{t}dt_{n}\;j\left(x_{n},t_{n}-t_{n-1}|x_{n-1}\right)S\left(t-t_{n}|x_{n}\right), \end{array}$$

where

(8) $$S\left(t|x_{0}\right)=\int_{t}^{\infty }dt^{\prime }\int_{\Gamma }dx\;j\left(x,t^{\prime }|x_{0}\right)$$

is the survival probability of the molecule up to time *t*, i.e., the probability of no adsorption up to time *t*. Indeed, the expression (7) describes the molecule that was adsorbed for the first time at time $t_{1}$ at point $x_{1}\in \Gamma$, then desorbed immediately, diffused in Ω, re-adsorbed at time $t_{2}$ at point $x_{2}\in \Gamma$, etc., until the last adsorption at time $t_{n}$ at point $x_{n}\in \Gamma$. The Laplace transform allows one to get rid of convolutions in time, yielding

(9) $$\begin{array}{cc} {\tilde{Q}}_{n}\left(p|x_{0}\right) & =\int_{0}^{\infty }dt\;e^{-pt}\;Q_{n}\left(t|x_{0}\right)\\ \; & =\int_{\Gamma }dx_{1}\;\tilde{j}\left(x_{1},p|x_{0}\right)\int_{\Gamma }dx_{2}\;\tilde{j}\left(x_{2},p|x_{1}\right)\ldots \int_{\Gamma }dx_{n}\;\tilde{j}\left(x_{n},p|x_{n-1}\right)\;\tilde{S}\left(p|x_{n}\right). \end{array}$$

However, convolutions on the boundary (i.e., the integrals over $x_{1},\ldots ,x_{n}\in \Gamma$) are difficult to deal with by conventional tools such as spectral expansions over Laplacian eigenfunctions [1]. In contrast, one can rely on spectral expansions based on the Steklov eigenfunctions [84]. Indeed, the Laplace transform of the encounter propagator reads [74]

(10) $$\begin{array}{cc} \tilde{P}\left(x,\ell ,p|x_{0}\right) & ={\tilde{G}}_{\infty }\left(x,p|x_{0}\right)\delta \left(\ell \right)+\frac{1}{D}\sum_{k=0}^{\infty }{\left[V_{k}^{\left(p\right)}\left(x_{0}\right)\right]}^{*}V_{k}^{\left(p\right)}\left(x\right)e^{-\mu_{k}^{\left(p\right)}\ell }, \end{array}$$

where asterisk denotes the complex conjugate, δ(ℓ) is the Dirac distribution, and ${\tilde{G}}_{\infty }\left(x,p|x_{0}\right)$ is Green’s function of the modified Helmholtz equation with mixed Dirichlet–Neumann boundary conditions:

(11a) $$\begin{array}{cc} \left(p-D\Delta \right){\tilde{G}}_{\infty }\left(x,p|x_{0}\right) & =\delta \left(x-x_{0}\right)\;\left(x\in \Omega \right), \end{array}$$

(11b) $$\begin{array}{cc} {\tilde{G}}_{\infty }\left(x,p|x_{0}\right) & =0\;(x\in \Gamma ), \end{array}$$

(11c) $$\begin{array}{cc} \partial_{n}{\tilde{G}}_{\infty }\left(x,p|x_{0}\right) & =0\;(x\in \partial \Omega_{0}), \end{array}$$

and $\mu_{k}^{\left(p\right)}$ and $V_{k}^{\left(p\right)}$ are the eigenvalues and eigenfunctions of the Steklov–Neumann spectral problem:

(12a) $$\begin{array}{cc} \left(p-D\Delta \right)V_{k}^{\left(p\right)} & =0\;\mathrm{in}\;\Omega , \end{array}$$

(12b) $$\begin{array}{cc} \partial_{n}V_{k}^{\left(p\right)} & =\mu_{k}^{\left(p\right)}V_{k}^{\left(p\right)}\;\mathrm{on}\;\Gamma , \end{array}$$

(12c) $$\begin{array}{cc} \partial_{n}V_{k}^{\left(p\right)} & =0\;\mathrm{on}\;\partial \Omega_{0}. \end{array}$$

When the boundary Γ is bounded, the spectrum of the Steklov problem is discrete, the eigenvalues can be ordered in an increasing sequence, $0\leq \mu_{0}^{\left(p\right)}\leq \mu_{1}^{\left(p\right)}\leq \ldots ↗+\infty$, whereas the restrictions $v_{k}^{\left(p\right)}=V_{k}^{\left(p\right)}{|}_{\Gamma }$ of Steklov eigenfunctions onto the adsorbing boundary Γ form a complete orthonormal basis of the space $L^{2}\left(\Gamma \right)$ of square-integrable functions [84,85,86].

After simplifications, the spectral expansion (10) implies

(13) $$\tilde{j}\left(x,p|x_{0}\right)=\sum_{k=0}^{\infty }{\left[V_{k}^{\left(p\right)}\left(x_{0}\right)\right]}^{*}v_{k}^{\left(p\right)}\left(x\right)\;\Upsilon \left(\mu_{k}^{\left(p\right)}\right),$$

where

(14) $$\Upsilon \left(\mu \right)=E\left\{e^{-\mu \hat{\ell }}\right\}=\int_{0}^{\infty }d\ell \;e^{-\mu \ell }\;\left(-\partial_{\ell }\Psi \left(\ell \right)\right)$$

is the moment-generating function of the threshold $\hat{\ell }$, with $\left(-\partial_{\ell }\Psi \left(\ell \right)\right)$ being its probability density function (PDF). In particular, Equation (8) implies

(15) $$\tilde{S}\left(p|x_{0}\right)=\frac{1}{p}-\frac{1}{p}\sum_{k=0}^{\infty }\Upsilon \left(\mu_{k}^{\left(p\right)}\right)c_{k}^{\left(p\right)}\left(x_{0}\right),$$

where

(16) $$c_{k}^{\left(p\right)}\left(x_{0}\right)={\left[V_{k}^{\left(p\right)}\left(x_{0}\right)\right]}^{*}\int_{\Gamma }v_{k}^{\left(p\right)}\left(x\right).$$

Substituting the spectral expansions (13) and (15) into Equation (9), one can use the orthogonality of $\left\{v_{k}^{\left(p\right)}\right\}$ to obtain

(17a) $$\begin{array}{cc} {\tilde{Q}}_{0}\left(p|x_{0}\right) & =\frac{1}{p}-{\tilde{R}}_{1}\left(p|x_{0}\right), \end{array}$$

(17b) $$\begin{array}{cc} {\tilde{Q}}_{n}\left(p|x_{0}\right) & ={\tilde{R}}_{n}\left(p|x_{0}\right)-{\tilde{R}}_{n+1}\left(p|x_{0}\right), \end{array}$$

where

(18) $${\tilde{R}}_{n}\left(p|x_{0}\right)=\frac{1}{p}\sum_{k=0}^{\infty }{\left[\Upsilon \left(\mu_{k}^{\left(p\right)}\right)\right]}^{n}\;c_{k}^{\left(p\right)}\left(x_{0}\right).$$

Even though this expression remains rather formal, it constitutes the main result of this paper that lays the theoretical ground for further analysis of the statistics of adsorptions. In fact, it incorporates the effect of the geometrical structure of the confining domain Ω through the Steklov eigenvalues and eigenfunctions, and the adsorption mechanism through the moment-generating function Υ(μ) of the threshold $\hat{\ell }$. When the molecule has a random lifetime τ obeying the exponential distribution with the rate *p*, Equation (17) determines the statistics of adsorption events realized during the lifetime, $N_{\tau }$, in a rather explicit form. In turn, the inverse Laplace transform of Equation (17) is needed to access the statistics $Q_{n}\left(t|x_{0}\right)$ of $N_{t}$.

One can also deduce the positive integer-order moments of the number of adsorptions. For instance, the mean number of adsorption events,

(19) $$N_{1}\left(t|x_{0}\right)=E_{x_{0}}\left\{N_{t}\right\}=\sum_{n=1}^{\infty }n\;Q_{n}\left(t|x_{0}\right),$$

reads in the Laplace domain as

(20) $${\tilde{N}}_{1}\left(p|x_{0}\right)=\frac{1}{p}\sum_{k=0}^{\infty }c_{k}^{\left(p\right)}\left(x_{0}\right){\left(\frac{1}{\Upsilon (\mu_{k}^{\left(p\right)})}-1\right)}^{-1}.$$

Similarly, one obtains the second moment

(21) $$\begin{array}{cc} {\tilde{N}}_{2}\left(p|x_{0}\right)\; & ={\tilde{N}}_{1}\left(p|x_{0}\right)+\frac{2}{p}\sum_{k=0}^{\infty }c_{k}^{\left(p\right)}\left(x_{0}\right){\left(\frac{1}{\Upsilon (\mu_{k}^{\left(p\right)})}-1\right)}^{-2}. \end{array}$$

As stated earlier, the above analysis assumes that the adsorbed molecule is immediately released. It is easy to incorporate a random duration $\delta_{k}$ in the adsorbed state. Let ϕ(t) denote the probability density function of random durations $\delta_{1},\ldots ,\delta_{n}$, which are assumed to be independent from each other and from the diffusive dynamics. In this case, *n* additional convolutions with ϕ(t) should be included into Equation (7) that results in the additional factor ${\left[\tilde{\phi }\left(p\right)\right]}^{n}$ in Equation (17b). The presence of additional waiting times in the adsorbed states delays the growth of the number of absorption events with *t*. While this effect can be important in practice, its implementation is straightforward and thus omitted here; in other words, we restrict our analysis to infinitely short absorption events and thus ignore $\tilde{\phi }\left(p\right)$ in the following analysis.

It is also instructive to recall that the Laplace transform of the mean boundary local time, $L\left(t|x_{0}\right)=E_{x_{0}}\left\{\ell_{t}\right\}$, admits a similar but much simpler form [80]:

(22) $$\tilde{L}\left(p|x_{0}\right)=\frac{1}{p}\sum_{k=0}^{\infty }\frac{c_{k}^{\left(p\right)}\left(x_{0}\right)}{\mu_{k}^{\left(p\right)}}\;.$$

Comparing Equations (20) and (22), one sees how the moment-generating function Υ(μ) of the threshold $\hat{\ell }$ affects each term of the spectral expansion in Equation (22) to transform the mean boundary local time (i.e., the mean number of adsorption attempts) into the mean number of adsorptions (i.e., successful outcomes of these attempts). One may naturally wonder under which condition these two quantities are proportional to each other, i.e., a prescribed fraction of adsorption attempts is successful? Setting the proportionality condition

(23) $$N_{1}\left(t|x_{0}\right)=qL\left(t|x_{0}\right)$$

with a parameter q>0, one immediately obtains

(24) $$\Upsilon \left(\mu_{k}^{\left(p\right)}\right)=\frac{1}{1+\mu_{k}^{\left(p\right)}/q}$$

for all $\mu_{k}^{\left(p\right)}$, i.e., one obtains the exponential law $\Psi \left(\ell \right)=e^{-q\ell }$ for the random threshold $\hat{\ell }$. In other words, the exponential law of $\hat{\ell }$ is the necessary and sufficient condition for getting a constant fraction of successful adsorption events among all attempts. As discussed in [74], this setting of a constant reactivity κ=qD is usually characterized by the Robin boundary condition on the target Γ.

The proportionality relation (23) can also be seen as a macroscopic definition of the constant reactivity κ via the ratio between the mean number of adsorptions (multiplied by *D*) and the mean boundary local time time. This relation generalizes a recent result by Kay and Giuggioli, which was derived for permeation dynamics of one-dimensional diffusion on the real line [87]. They suggested to consider the ratio between the mean number of permeation events and the mean boundary local time as a macroscopic definition of permeability. We make a step further and introduce the effective macroscopic reactivity of the target Γ for an arbitrary adsorption kinetics as

(25) $$\kappa \left(t|x_{0}\right)=D\frac{N_{1}\left(t|x_{0}\right)}{L(t|x_{0})}\;.$$

When the threshold $\hat{\ell }$ exhibits an exponential distribution, this definition yields a constant reactivity κ according to Equation (23). However, when the probability law Ψ(ℓ) of the threshold $\hat{\ell }$ is not exponential, the ratio in Equation (25) may exhibit a non-trivial dependence on both *t* and $x_{0}$, as illustrated in Section 4.

### 2.2. Long-Time Behavior for Bounded Domains

The inversion of the Laplace transform can be performed via the residue theorem by identifying the poles of the function ${\tilde{Q}}_{n}\left(p|x_{0}\right)$ in the complex plane p∈C. However, this analysis is rather subtle and requires the knowledge of the Steklov eigenvalues and eigenfunctions for a given confining domain Ω. For the sake of clarify, we skip this analysis and focus on the long-time behavior of the statistics of adsorption events, which corresponds to the limit p→0 in the Laplace domain. This behavior strongly depends on whether the confining domain Ω is bounded or not. In this section, we consider bounded domains, while the case of unbounded domains is briefly discussed in Section 2.3.

When Ω is bounded, the smallest eigenvalue $\mu_{0}^{\left(p\right)}$ approaches $\mu_{0}^{\left(0\right)}=0$ as p→0, while the corresponding eigenfunction $V_{0}^{\left(p\right)}$ approaches $V_{0}^{\left(0\right)}=1/\sqrt{\left|\Gamma \right|}$, where |Γ| is the surface area of the adsorbing boundary Γ. In fact, one has [80,88]

(26) $$\mu_{0}^{\left(p\right)}\approx \frac{p|\Omega |}{D|\Gamma |}+O\left(p^{2}\right)\;\left(p\to 0\right),$$

where |Ω| is the volume of the confining domain. In turn, all other eigenvalues $\mu_{k}^{\left(p\right)}$ approach their strictly positive limits $\mu_{k}^{\left(0\right)}>0$ for k=1,2,…. Moreover, since the eigenfunctions $v_{k}^{\left(0\right)}$ are orthogonal to $v_{0}^{\left(0\right)}=1/\sqrt{\left|\Gamma \right|}$, one has

(27) $$c_{k}^{\left(p\right)}\left(x_{0}\right)=\delta_{k,0}+O\left(p\right)\;\left(p\to 0\right),$$

where $\delta_{j,k}$ is the Kronecker symbol.

Next, we consider a general asymptotic behavior of the moment-generating function,

(28) $$\Upsilon \left(\mu \right)\approx 1-{\left(\mu \ell_{0}\right)}^{\alpha }+o\left(\mu^{\alpha }\right)\;\left(\mu \to 0\right),$$

with a length scale $\ell_{0}$ and an exponent 0<α≤1. Since the mean threshold can be obtained from the moment-generating function as $E\left\{\hat{\ell }\right\}=-\lim_{\mu \to 0}\partial_{\mu }\Upsilon \left(\mu \right)$, the exponent α distinguishes two cases when the mean threshold is finite (α=1) or infinite (α<1).

#### 2.2.1. Mean and Standard Deviation

Substituting Equations (26) and (28) into the first term in Equations (20) and (21) and evaluating the leading-order terms, we obtain the long-time behavior for any 0<α≤1:

(29a) $$\begin{array}{cc} N_{1}\left(t|x_{0}\right) & \approx \frac{{\left(t/t_{0}\right)}^{\alpha }}{\Gamma (1+\alpha )}+O\left(1\right), \end{array}$$

(29b) $$\begin{array}{cc} N_{2}\left(t|x_{0}\right) & \approx \frac{2{\left(t/t_{0}\right)}^{2\alpha }}{\Gamma (1+2\alpha )}+O\left({\left(t/t_{0}\right)}^{\alpha }\right), \end{array}$$

where $t_{0}=\ell_{0}\left|\Omega \right|/\left(D|\Gamma |\right)$. As a consequence, the standard deviation of $N_{t}$, defined as $\Delta N_{t}=\sqrt{N_{2}\left(t|x_{0}\right)-{\left[N_{1}\left(t|x_{0}\right)\right]}^{2}}$, also exhibits a power-law growth at long times. However, there is a significant difference between cases α<1 and α=1. In the former case, one obtains

(30) $$\Delta N_{t}\approx \sqrt{\frac{2}{\Gamma (1+2\alpha )}-\frac{1}{\Gamma^{2}\left(1+\alpha \right)}}\;{\left(t/t_{0}\right)}^{\alpha }\;\left(t\to \infty \right),$$

i.e., the standard deviation of $N_{t}$ grows in the same way as its mean value. In other words, the coefficient of variation, $\Delta N_{t}/N_{1}\left(t|x_{0}\right)$, approaches a constant at long times. In contrast, if α=1, the coefficient in front of ${\left(t/t_{0}\right)}^{\alpha }$ vanishes, and the standard deviation is determined by the next-order term, yielding $\Delta N_{t}\propto {\left(t/t_{0}\right)}^{1/2}$. In this case, the coefficient of variation vanishes, i.e., the statistics of adsorption events becomes more concentrated around the mean value.

On the other hand, the mean boundary local time exhibits the long-time behavior [80]:

(31) $$L\left(t|x_{0}\right)\approx \frac{Dt|\Gamma |}{\left|\Omega \right|}\;\left(t\to \infty \right).$$

Substituting these asymptotic relations into Equation (25), we obtain the following long-time behavior of the effective reactivity:

(32) $$\kappa \left(t|x_{0}\right)\approx \frac{D}{\ell_{0}}\;\frac{{\left(t/t_{0}\right)}^{\alpha -1}}{\Gamma (1+\alpha )}\;\left(t\to \infty \right).$$

This relation further clarifies the effect of the adsorption mechanism through the probability law of the random threshold $\hat{\ell }$:

(i) If the mean threshold $E\{\hat{\ell }\}$ is finite (and thus equal to $\ell_{0}$), one has α=1 and thus, the effective reactivity reaches a constant value $D/\ell_{0}$. As a consequence, the effects of a non-exponential threshold can only emerge at short and intermediate times, at which $\kappa (t|x_{0})$ may exhibit non-trivial dependence on *t* and $x_{0}$, but the long-time asymptotic behavior remains similar to the conventional setting with the exponential law (with the only difference that the reactivity parameter *q* is replaced by $1/\ell_{0}$).

(ii) In contrast, if the mean threshold $E\{\hat{\ell }\}$ is infinite (and thus α<1), the effective reactivity asymptotically vanishes with time. In fact, a heavy-tailed distribution of the threshold allows to produce anomalously large values of $\hat{\ell }$ that require long times to achieve the adsorption condition $\ell_{t}>\hat{\ell }$. Moreover, as the adsorption events are repeated many times in the limit t→∞, larger and larger thresholds ${\hat{\ell }}_{n}$ can be generated and yield longer and longer first-crossing times $T_{n}$ so that the fraction of successful adsorptions decreases with time. The effect of such non-Markovian binding mechanism onto diffusion-controlled reactions was discussed in [77].

#### 2.2.2. Distribution for the Case α<1

Separating the first term with k=0 in Equation (18) from the other terms and substituting Equations (26) and (28) into this first term, we obtain in the limit p→0

(33) $${\tilde{R}}_{n}\left(p|x_{0}\right)\approx \frac{1}{p}{\left(1-{\left(pt_{0}\right)}^{\alpha }\right)}^{n},$$

where we used Equation (27) to cancel all the terms with k>0. Substituting this expression into Equation (17) and applying the inverse Laplace transform, we obtain the leading order for any 0<α<1:

(34) $$Q_{n}\left(t|x_{0}\right)\approx \frac{{\left(t/t_{0}\right)}^{-\alpha }}{\Gamma (1-\alpha )}\;\left(t\to \infty \right)$$

for any fixed n=0,1,2,…. One sees that the probability of having exactly *n* adsorption events slowly vanishes with time. Note that obtaining the opposite limit n→∞ for a fixed *t* is a more difficult task (see an example in Section 4.1), and these two limits are not interchangeable.

#### 2.2.3. Distribution for the Case α=1

A naive extension of the above analysis to the case α=1 is not valid. We therefore sketch a different approach based on the Laplace transform inversion via the Bromwich integral. Indeed, the long-time asymptotic behavior of $Q_{n}\left(t|x_{0}\right)$ is determined by the pole $p_{0}<0$ of ${\tilde{Q}}_{n}\left(p|x_{0}\right)$ with the smallest absolute value $|p_{0}|$. The asymptotic behavior would thus be exponential in the leading order, i.e., $Q_{n}\left(t|x_{0}\right)\propto e^{p_{0}t}$, with the prefactor being a polynomial of *t*, which depends on *n*. The value of $p_{0}$ and the form of the prefactor strongly depend on the moment-generating function Υ(μ) and the Steklov eigenvalues $\mu_{k}^{\left(p\right)}$. Their detailed analysis is beyond the scope of this paper.

### 2.3. Long-Time Behavior for Unbounded Domains

In many situations, one deals with a compact target in the space $R^{d}$, i.e., the confining domain $\Omega =R^{d}\setminus \Omega_{0}$ is the exterior of a compact set $\Omega_{0}$. Since the confining domain is now unbounded, conventional spectral expansions over the eigenmodes of the Laplace operator are not suitable. In contrast, one can still employ the Steklov eigenmodes since the underlying eigenvalue problem (12) has a discrete spectrum. The asymptotic analysis is more subtle and strongly depends on the space dimension *d*. Indeed, the one-dimensional case (a half-line) is elementary (see Section 4.1) and presents a rare example when most results can be derived in an explicit form. The distribution of the boundary local time for planar domains was studied in [89]. While the developed tools can potentially be applied to analyze the statistics of adsorption events, their thorough discussion is beyond the scope of this paper.

In turn, the long-time asymptotic analysis of the three-dimensional setting, which is the most common for applications, is relatively simple. Indeed, as the principal eigenvalue $\mu_{0}^{\left(p\right)}$ approaches a strictly positive constant $\mu_{0}^{\left(0\right)}>0$, the spectral expansions (17) approach constant limits:

(35a) $$\begin{array}{cc} Q_{0}\left(\infty |x_{0}\right) & =1-\sum_{k=0}^{\infty }\Upsilon \left(\mu_{k}^{\left(0\right)}\right)\;c_{k}^{\left(0\right)}\left(x_{0}\right), \end{array}$$

(35b) $$\begin{array}{cc} Q_{n}\left(\infty |x_{0}\right) & =\sum_{k=0}^{\infty }\left(1-\Upsilon \left(\mu_{k}^{\left(0\right)}\right)\right){\left[\Upsilon \left(\mu_{k}^{\left(0\right)}\right)\right]}^{n}\;c_{k}^{\left(0\right)}\left(x_{0}\right). \end{array}$$

This asymptotic behavior is the consequence of the transient character of diffusion in three dimensions. Indeed, there is a strictly positive probability for the molecule to escape to infinity, in which case the statistics of adsorption events remains unchanged. We also stress that Equation (27), which we used for bounded domains, does not hold here so that (infinitely) many terms can in general contribute to these statistics. In this light, the usual case of a spherical target, for which only the principal eigenmode contributes (see Section 4.2), is an exception, not a rule. For instance, when the target has a spheroidal anisotropic shape, infinitely many terms do contribute, even though their relative contributions are progressively attenuated due to increasing eigenvalues $\mu_{k}^{\left(0\right)}$ [90]. Further analysis of this setting presents an interesting perspective of this work.

## 3. Statistics of Permeation Events

We now briefly discuss a related problem of finding the statistics of boundary crossings in the more challenging problem of a permeable interface between two confining domains (an extension to multiple confining domains with distinct permeable boundaries is also possible but not discussed here). Bressloff extensively applied the encounter-based approach to describe this phenomenon [41,42,76,91]. Let us consider two domains $\Omega_{1}$ and $\Omega_{2}$ with a common interface Γ. The molecule starts from a point $x_{0}\in \Omega_{1}$ and diffuses in $\Omega_{1}$ until an adsorption on Γ, as in the earlier considered case. However, the adsorbed molecule is now released on the other side of the interface Γ, namely, in $\Omega_{2}$. The diffusion continues until an adsorption on Γ and the consequent immediate release in $\Omega_{1}$, and so on. In this way, one can describe successive passages across the permeable interface Γ. As previously, we characterize the adsorption mechanisms on both sides via random thresholds which are now governed by two probability laws $\Psi_{1}\left(\ell \right)$ and $\Psi_{2}\left(\ell \right)$. One can therefore proceed in constructing the probability flux densities $j_{1}\left(x,t|x_{0}\right)$ and $j_{2}\left(x,t|x_{0}\right)$ in both domains. The number of permeation events $N_{t}$ can be defined as earlier, while its probability distribution is constructed in a similar way:

(36) $$\begin{array}{cc} Q_{0}\left(t|x_{0}\right) & =S_{1}\left(t|x_{0}\right), \end{array}$$

(37) $$\begin{array}{cc} Q_{2n}\left(t|x_{0}\right) & =\int_{\Gamma }dx_{1}\int_{0}^{t}dt_{1}\;j_{1}\left(x_{1},t_{1}|x_{0}\right)\int_{\Gamma }dx_{2}\int_{t_{1}}^{t}dt_{2}\;j_{2}\left(x_{2},t_{2}-t_{1}|x_{1}\right)\ldots \\ \; & \int_{\Gamma }dx_{2n}\int_{t_{2n-1}}^{t}dt_{2n}j_{2}\left(x_{2n},t_{2n}-t_{2n-1}|x_{2n-1}\right)S_{1}\left(t-t_{2n}|x_{2n}\right), \end{array}$$

(38) $$\begin{array}{cc} Q_{2n+1}\left(t|x_{0}\right) & =\int_{\Gamma }dx_{1}\int_{0}^{t}dt_{1}\;j_{1}\left(x_{1},t_{1}|x_{0}\right)\int_{\Gamma }dx_{2}\int_{t_{1}}^{t}dt_{2}\;j_{2}\left(x_{2},t_{2}-t_{1}|x_{1}\right)\ldots \\ \; & \int_{\Gamma }dx_{2n+1}\int_{t_{2n}}^{t}dt_{2n+1}j_{1}\left(x_{2n+1},t_{2n+1}-t_{2n}|x_{2n}\right)S_{2}\left(t-t_{2n+1}|x_{2n+1}\right), \end{array}$$

where we distinguished even and odd crossing numbers. As previously described, the Laplace transform reduces convolutions in time, whereas spectral expansions over Steklov eigenfunctions allow one to deal with convolutions on the boundary. However, the main difficulty here is that the Steklov eigenpairs, denoted as $\left\{\mu_{k}^{\left(p,1\right)},V_{k}^{\left(p,1\right)}\right\}$ and $\left\{\mu_{k}^{\left(p,2\right)},V_{k}^{\left(p,2\right)}\right\}$ in two domains, are different. As a consequence, the expressions for ${\tilde{Q}}_{n}\left(p|x_{0}\right)$ become more sophisticated, e.g.,

$$\begin{array}{cc} {\tilde{Q}}_{2n}\left(p|x_{0}\right) & =\frac{1}{p}\sum_{k_{1},k_{2},\ldots ,k_{2n},k_{2n+1}}{\left[V_{k_{1}}^{\left(p,1\right)}\left(x_{0}\right)\right]}^{*}\Upsilon_{1}\left(\mu_{k_{1}}^{\left(p,1\right)}\right)W_{k_{1},k_{2}}\Upsilon_{2}\left(\mu_{k_{2}}^{\left(p,2\right)}\right)W_{k_{2},k_{3}}^{†}\ldots \\ \; & \times W_{k_{2n-1},k_{2n}}^{†}\Upsilon_{2}\left(\mu_{k_{2n}}^{\left(p,2\right)}\right)\left(\delta_{k_{2n},k_{2n+1}}-W_{k_{2n},k_{2n+1}}\Upsilon_{1}\left(\mu_{k_{2n+1}}^{\left(p,1\right)}\right)\right)\int_{\Gamma }v_{k_{2n+1}}^{\left(p,1\right)}, \end{array}$$

where

(39) $$W_{k,k^{\prime }}=\int_{\Gamma }v_{k}^{\left(p,1\right)}\;{\left[v_{k^{\prime }}^{\left(p,2\right)}\right]}^{*}$$

(a similar expression holds for ${\tilde{Q}}_{2n+1}\left(p|x_{0}\right)$, while ${\tilde{Q}}_{0}\left(p|x_{0}\right)$ is equal to ${\tilde{S}}_{1}\left(p|x_{0}\right)$). The matrix structure of the above expression allows for a rapid numerical evaluation of ${\tilde{Q}}_{2n}\left(p|x_{0}\right)$, but its theoretical analysis becomes challenging.

### Spherical Domains

A considerable simplification appears in the special case when the two sets of eigenfunctions $\left\{v_{k}^{\left(p,1\right)}\right\}$ and $\left\{v_{k}^{\left(p,2\right)}\right\}$ on the target Γ are identical. For instance, if $\Omega_{1}$ is a ball of radius *R* and $\Omega_{2}$ is a shell between two concentric spheres of radii *R* and *L*, the eigenfunctions $v_{k}^{\left(p,1\right)}$ and $v_{k}^{\left(p,2\right)}$ are equal to spherical harmonics on the spherical interface Γ between two domains. In this case, *W* is the identity matrix, and the above expressions greatly simplify as

(40a) $$\begin{array}{cc} {\tilde{Q}}_{0}\left(p|x_{0}\right) & =\frac{1}{p}-\frac{1}{p}\sum_{k=0}^{\infty }\Upsilon_{1}\left(\mu_{k}^{\left(p,1\right)}\right)c_{k}^{\left(p\right)}\left(x_{0}\right), \end{array}$$

(40b) $$\begin{array}{cc} {\tilde{Q}}_{2n}\left(p|x_{0}\right) & =\frac{1}{p}\sum_{k=0}^{\infty }{\left[\Upsilon_{1}\left(\mu_{k}^{\left(p,1\right)}\right)\Upsilon_{2}\left(\mu_{k}^{\left(p,2\right)}\right)\right]}^{n}\left(1-\Upsilon_{1}\left(\mu_{k}^{\left(p,1\right)}\right)\right)c_{k}^{\left(p\right)}\left(x_{0}\right), \end{array}$$

(40c) $$\begin{array}{cc} {\tilde{Q}}_{2n+1}\left(p|x_{0}\right) & =\frac{1}{p}\sum_{k=0}^{\infty }{\left[\Upsilon_{1}\left(\mu_{k}^{\left(p,1\right)}\right)\Upsilon_{2}\left(\mu_{k}^{\left(p,2\right)}\right)\right]}^{n}\Upsilon_{1}\left(\mu_{k}^{\left(p,1\right)}\right)\left(1-\Upsilon_{2}\left(\mu_{k}^{\left(p,2\right)}\right)\right)c_{k}^{\left(p\right)}\left(x_{0}\right), \end{array}$$

where

(41) $$\begin{array}{cc} c_{k}^{\left(p\right)}\left(x_{0}\right) & ={\left[V_{k}^{\left(p,1\right)}\left(x_{0}\right)\right]}^{*}\int_{\Gamma }v_{k}^{\left(p,1\right)}. \end{array}$$

A lengthy but elementary calculation yields the mean number of permeation events in the Laplace domain

(42) $${\tilde{N}}_{1}\left(p|x_{0}\right)=\frac{1}{p}\sum_{k=0}^{\infty }\frac{\Upsilon_{1}\left(\mu_{k}^{\left(p,1\right)}\right)\left[1+\Upsilon_{2}\left(\mu_{k}^{\left(p,2\right)}\right)\right]}{1-\Upsilon_{1}\left(\mu_{k}^{\left(p,1\right)}\right)\Upsilon_{2}\left(\mu_{k}^{\left(p,2\right)}\right)}c_{k}^{\left(p\right)}\left(x_{0}\right).$$

If the domains and adsorption mechanisms were identical, i.e., if $\mu_{k}^{\left(p,1\right)}=\mu_{k}^{\left(p,2\right)}=\mu_{k}^{\left(p\right)}$ and $\Upsilon_{1}\left(\mu \right)=\Upsilon_{2}\left(\mu \right)=\Upsilon \left(\mu \right)$, the above expression would reduce to Equation (20), as it should.

As the long-time behavior corresponds to the limit p→0, one needs to distinguish bounded and unbounded domains. In the former case, one has $\mu_{0}^{\left(p,i\right)}\approx p|\Omega_{i}\left|/(D|\Gamma |\right)$. Substituting this expression into

(43) $$\Upsilon_{i}\left(\mu \right)=1-{\left(\mu \ell_{i}\right)}^{\alpha_{i}}+o\left(\mu^{\alpha_{i}}\right)\;\left(\mu \to 0\right),$$

we obtain in the leading order

(44) $${\tilde{N}}_{1}\left(p|x_{0}\right)\approx \frac{2}{p[{\left(pt_{1}\right)}^{\alpha_{1}}+{\left(pt_{2}\right)}^{\alpha_{2}}]}\;\left(p\to 0\right)\;,$$

where $t_{i}=\ell_{i}|\Omega_{i}\left|/(D|\Gamma |\right)$. If $\alpha_{1}=\alpha_{2}=\alpha$, one finds

(45) $$N_{1}\left(t|x_{0}\right)\approx \frac{2t^{\alpha }}{\Gamma \left(1+\alpha \right)\left[t_{1}^{\alpha }+t_{2}^{\alpha }\right]}\;\left(t\to \infty \right).$$

If $\alpha_{1}<\alpha_{2}$, the slower adsorption mechanism from $\Omega_{1}$ on Γ determines the long-time asymptotic behavior:

(46) $$N_{1}\left(t|x_{0}\right)\approx \frac{2t^{\alpha_{1}}}{\Gamma \left(1+\alpha_{1}\right)t_{1}^{\alpha_{1}}}\;\left(t\to \infty \right).$$

In turn, if one of two domains is unbounded (say, $\Omega_{2}$), all eigenvalues $\mu_{k}^{\left(p,2\right)}$ approach strictly positive limits $\mu_{k}^{\left(0,2\right)}$ in three dimensions so that the leading term is different, ${\tilde{N}}_{1}\left(p|x_{0}\right)=O\left(1/p\right)$, yielding another long-time asymptotic behavior

(47) $$N_{1}\left(\infty |x_{0}\right)=\sum_{k=0}^{\infty }\frac{\Upsilon_{1}\left(\mu_{k}^{\left(0,1\right)}\right)\left[1+\Upsilon_{2}\left(\mu_{k}^{\left(0,2\right)}\right)\right]}{1-\Upsilon_{1}\left(\mu_{k}^{\left(0,1\right)}\right)\Upsilon_{2}\left(\mu_{k}^{\left(0,2\right)}\right)}c_{k}^{\left(0\right)}\left(x_{0}\right).$$

The constant limit of $N_{1}\left(t|x_{0}\right)$ reflects the transient diffusion in the unbounded domain $\Omega_{2}$, i.e., an eventual escape of the molecule to infinity.

## 4. Two Relevant Examples

While our formalism allows one to investigate the statistics of adsorption and permeation events in arbitrary environments, its application requires a numerical computation of the Steklov eigenmodes and inverse Laplace transform. Even though various numerical methods are available (see [92] and references therein), we focus here on two practically relevant examples: a flat surface (Section 4.1) and a spherical surface (Section 4.2). In both cases, the Steklov eigenmodes are known explicitly to yield closed expressions for the distributions. It is worth noting that the overwhelming majority of earlier studies of adsorption phenomena and reversible binding reactions were also limited to these two settings (see [77,78] and references therein).

### 4.1. Flat Surface

We first consider molecular diffusion in the upper half-space bounded by an adsorbing horizontal plane. As lateral displacements of the molecule do not affect adsorption events, this problem is equivalent to diffusion on the positive half-line, Ω=(0,+∞), for which the Steklov problem has a single eigenpair,

(48) $$\mu_{0}^{\left(p\right)}=\sqrt{p/D},\;V_{0}^{\left(p\right)}\left(x\right)=e^{-x\sqrt{p/D}},$$

so that $c_{0}^{\left(p\right)}\left(x_{0}\right)=e^{-x_{0}\sqrt{p/D}}$ from Equation (16) and thus,

(49a) $$\begin{array}{cc} {\tilde{Q}}_{0}\left(p|x_{0}\right) & =\frac{1}{p}-\frac{e^{-x_{0}\sqrt{p/D}}}{p}\Upsilon \left(\sqrt{p/D}\right), \end{array}$$

(49b) $$\begin{array}{cc} {\tilde{Q}}_{n}\left(p|x_{0}\right) & =\frac{e^{-x_{0}\sqrt{p/D}}}{p}{\left[\Upsilon \left(\sqrt{p/D}\right)\right]}^{n}\left[1-\Upsilon \left(\sqrt{p/D}\right)\right]. \end{array}$$

**Figure 2:**
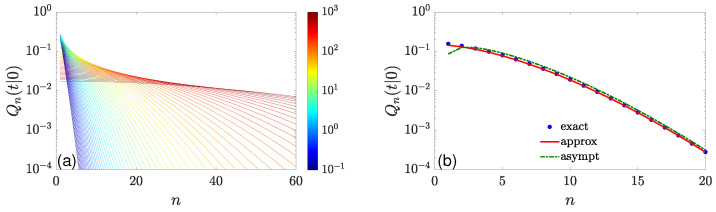
Statistics of adsorption events at the origin for diffusion on a half-line with a constant reactivity q=1. (**a**) $Q_{n}\left(t|0\right)$ as a function of *n* for 64 values of *t*, chosen equidistantly on a logarithmic scale, from $t=10^{-1}$ (dark blue) to $t=10^{3}$ (dark red), with D=1. (**b**) $Q_{n}\left(t|0\right)$ as a function of *n* for t=10. Filled circles present the exact solution (50), the solid line shows the approximation (52), whereas the dash-dotted line indicates the asymptotic relation (53).

For the case of a constant reactivity and $x_{0}=0$, one can substitute $\Upsilon \left(\sqrt{p/D}\right)=1/\left(1+\sqrt{p/D}/q\right)$ into this expression and then invert the Laplace transform to represent $Q_{n}\left(t|0\right)$ in terms of the parabolic cylinder function (see [93], p. 43, Equation (21)). After simplifications, we obtain

(50) $$Q_{n}\left(t|0\right)=\frac{1}{\sqrt{\pi }}{\left(q^{2}Dt\right)}^{n/2}\;U\left(\left(n+1\right)/2,1/2,q^{2}Dt\right),$$

where U(a,b,z) is the Tricomi confluent hypergeometric function. We therefore retrieved the result by Kay and Giuggioli [87]. Note that $Q_{0}\left(t|0\right)=\mathrm{erfcx}\left(q\sqrt{Dt}\right)$, where $\mathrm{erfcx}\left(z\right)=e^{z^{2}}\mathrm{erfc}\left(z\right)$ is the scaled complementary error function (moreover, $Q_{0}\left(t|x_{0}\right)$ can be written explicitly for any $x_{0}>0$). Figure 2a illustrates the dependence of $Q_{n}\left(t|0\right)$ of *n* for a broad range of times.

Since $U\left(a,b,z\right)\approx z^{-a}$ as z→∞, we obtain

(51) $$Q_{n}\left(t|0\right)\approx \frac{1}{\sqrt{\pi }}{\left(q^{2}Dt\right)}^{-1/2}\;\left(t\to \infty \right).$$

The long-time decay of these probabilities is very slow and independent of *n*.

In turn, the opposite limit of large *n* can be obtained from the asymptotic behavior of U(a,b,z) for large *a* (see [94]) that yields

(52) $$Q_{n}\left(t|0\right)\approx \frac{2z^{n/2}e^{z/2-\beta (n+1)}}{\sqrt{n+1}\;\Gamma \left(\left(n+1\right)/2\right)\sqrt{1+e^{-w}}}\;,$$

where $z=q^{2}Dt$, w=arccosh(1+z/(n+1)), and β=(w+sinhw)/2. While the dependence on *n* is somewhat cumbersome here, this asymptotic relation turns out to be remarkably accurate for all *t* (Figure 2b). When n≫z, one obtains $\beta \approx w\approx \sqrt{2z/n}\ll 1$ so that

(53) $$Q_{n}\left(t|0\right)\approx \frac{z^{n/2}e^{z/2-\sqrt{2nz}-\sqrt{2z/n}}}{\sqrt{n/2}\;\Gamma \left(n/2\right)}\;,$$

and the factor Γ(n/2) provides faster-than-exponential decay of $Q_{n}\left(t|0\right)$ as n→∞.

One can also compute the moments of the number of adsorption events; for instance, the spectral expansion in Equation (20) contains a single term, and its inverse Laplace transform yields $N_{1}\left(t|x_{0}\right)=qL\left(t|x_{0}\right)$, with

(54) $$L\left(t|x_{0}\right)=\frac{\sqrt{4Dt}}{\sqrt{\pi }}e^{-x_{0}^{2}/\left(4Dt\right)}-x_{0}\;\mathrm{erfc}\left(x_{0}/\sqrt{4Dt}\right).$$

At $x_{0}=0$, one obtains $N_{1}\left(t|0\right)=2q\sqrt{Dt}/\sqrt{\pi }$, which is also the long-time asymptotic behavior for any $x_{0}\geq 0$. This sublinear growth with time is a consequence of the unboundedness of the half-line: the molecule can diffuse far away from the origin to keep the number of adsorption events constant for long periods. In contrast, $N_{1}\left(t|x_{0}\right)$ grows linearly with time for any bounded domain at long times, see Equation (29a).

Since the spectrum of the Steklov problem is particularly simple for a flat surface, one can determine the statistics of the adsorption events for any adsorption mechanism defined via a given moment-generating function Υ(μ). To illustrate its effect, we choose a simple model when the molecule adsorbs after a fixed amount of attempts. This deterministic threshold, $\hat{\ell }=\ell_{0}$, with some $\ell_{0}>0$, corresponds to $\Upsilon \left(\mu \right)=e^{-\mu \ell_{0}}$. Substituting this expression into Equation (49) and performing the inverse Laplace transform, we obtain

(55a) $$\begin{array}{cc} Q_{0}\left(t|x_{0}\right) & =1-\mathrm{erfc}\left(\frac{x_{0}+\ell_{0}}{\sqrt{4Dt}}\right), \end{array}$$

(55b) $$\begin{array}{cc} Q_{n}\left(t|x_{0}\right) & =\mathrm{erfc}\left(\frac{x_{0}+n\ell_{0}}{\sqrt{4Dt}}\right)-\mathrm{erfc}\left(\frac{x_{0}+\left(n+1\right)\ell_{0}}{\sqrt{4Dt}}\right). \end{array}$$

In turn, the mean number of adsorptions is

(56) $$N_{1}\left(t|x_{0}\right)=\sum_{n=1}^{\infty }\mathrm{erfc}\left(\frac{x_{0}+n\ell_{0}}{\sqrt{4Dt}}\right).$$

**Figure 3:**
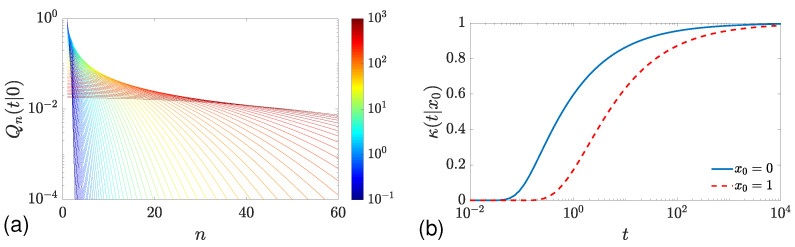
Statistics of adsorption events at the origin for diffusion on a half-line for the adsorption mechanism with a fixed threshold $\ell_{0}=1$. (**a**) $Q_{n}\left(t|0\right)$ as a function of *n* for 64 values of *t*, chosen equidistantly on logarithmic scale, from $t=10^{-1}$ (dark blue) to $t=10^{3}$ (dark red), with D=1. (**b**) The effective reactivity $\kappa (t|x_{0})$ defined in Equation (25), with $N_{1}\left(t|x_{0}\right)$ given by Equation (56) and $L(t|x_{0})$ given by Equation (54).

Figure 3a illustrates the statistics of adsorption events for a fixed threshold $\ell_{0}=1$. It is instructive to compare this panel with Figure 2a for a constant reactivity q=1. Even though the functional forms of $Q_{n}\left(t|0\right)$ in Equations (49) and (55) are different, these two statistics look similar at long times. This is not surprising as the exponentially distributed random threshold $\hat{\ell }$ has the mean value 1/q=1, which is identical to the fixed threshold $\ell_{0}=1$. In turn, some distinctions can be noticed at short times; in particular, $Q_{n}\left(t|0\right)$ decays faster with *n* when the threshold is fixed. The distinction at short times becomes even more explicit when plotting the effective reactivity $\kappa (t|x_{0})$ defined in Equation (25) as the ratio between the mean number of adsorptions, $N_{1}\left(t|x_{0}\right)$, and the mean boundary local time $L(t|x_{0})$. As this ratio is equal to 1 for a constant reactivity κ=qD=1, its deviations from 1 witness the effect of the non-exponential adsorption mechanism. Since the mean threshold is finite for the fixed threshold $\hat{\ell }=\ell_{0}$, the effectively reactivity $\kappa (t|x_{0})$ approaches $D/\ell_{0}=1$ at long times. In turn, $\kappa (t|x_{0})$ vanishes at short times. Indeed, as t→0, the molecule has almost no chance to hit the boundary enough times to exceed a fixed threshold $\hat{\ell }=\ell_{0}=1$. In turn, when the threshold $\hat{\ell }$ is exponentially distributed, its realized value can be much smaller, so that the adsorption at short times is more probable. We note also that if the starting point $x_{0}$ does not lie on the boundary (i.e., if $x_{0}>0$), the molecule needs first to reach the boundary that shifts the curve $N_{1}\left(t|x_{0}\right)$ to the right (to longer times). However, the starting point $x_{0}$ has no effect on the long-time asymptotic behavior, as expected.

Following [77], we also consider the Mittag–Leffler probability law for the threshold $\hat{\ell }$ as a model of anomalous reactivity:

(57) $$\Psi \left(\ell \right)=E_{\alpha }\left(-{\left(\ell /\ell_{0}\right)}^{\alpha }\right)\;\Rightarrow \;\Upsilon \left(\mu \right)=\frac{1}{1+{\left(\mu \ell_{0}\right)}^{\alpha }}\;,$$

with a length scale $\ell_{0}$ and an exponent 0<α≤1, where $E_{\alpha }\left(z\right)=\sum_{n=0}^{\infty }z^{n}/\Gamma \left(n+\alpha \right)$ is the Mittag–Leffler function. At α=1, one retrieves the exponential distribution with the mean threshold $\ell_{0}$. Substitution of Equation (57) into Equation (20) and the inversion of the Laplace transform with $x_{0}=0$ yield the exact result

(58) $$N_{1}\left(t|0\right)=\frac{{\left(Dt/\ell_{0}^{2}\right)}^{\alpha /2}}{\Gamma (1+\alpha /2)}\;.$$

Comparing this expression with $L\left(t|0\right)=2\sqrt{Dt}/\sqrt{\pi }$, we obtain the effective reactivity

(59) $$\kappa \left(t|0\right)=\frac{\left(D/\ell_{0}\right)\sqrt{\pi }}{2\Gamma (1+\alpha /2)}{\left(Dt/\ell_{0}^{2}\right)}^{(\alpha -1)/2}\;.$$

We stress that this exact formula is valid for any *t*. As discussed earlier in Section 2.2, the effective reactivity vanishes at long times. However, the exponent is twice smaller as compared to the case of bounded domains; see Equation (32). Curiously, the effective reactivity in Equation (59) diverges at short times. This counter-intuitive behavior is a consequence of the Mittag–Leffler model. In fact, the probability density of the threshold $\hat{\ell }$ behaves as $\left(-\partial_{\ell }\Psi \left(\ell \right)\right)\approx {\left(\ell /\ell_{0}\right)}^{\alpha -1}/\left(\ell_{0}\Gamma \left(\alpha \right)\right)$ as ℓ→0, i.e., it diverges for α<1. In contrast, the density would approach a constant value $1/\ell_{0}$ for α=1. The divergence for α<1 implies that small values of the threshold $\hat{\ell }$ are much more probable than in the case α=1. As a consequence, the adsorption events are much more frequent at short times, as if the boundary was extremely reactive. This argument rationalizes the divergence of the effective reactivity κ(t|0) as t→0 (see also a related discussion in [77]).

### 4.2. Spherical Surface

When $\Omega =\{x\in R^{3}\;:\;|x|<R\}$ is a ball of radius *R* with the adsorbing boundary Γ=∂Ω, the Steklov eigenvalue problem admits an explicit solution [75,84]. In spherical coordinates x=(r,θ,ϕ), one has

(60) $$\mu_{k}^{\left(p\right)}=\sqrt{p/D}\;\frac{i_{k}^{\prime }\left(R\sqrt{p/D}\right)}{i_{k}\left(R\sqrt{p/D}\right)},\;V_{k}^{\left(p\right)}=\frac{i_{k}\left(r\sqrt{p/D}\right)}{i_{k}\left(R\sqrt{p/D}\right)}Y_{k,m}\left(\theta ,\phi \right),$$

where prime denotes the derivative with respect to the argument, $i_{n}\left(z\right)=\sqrt{\pi /(2z)}I_{n+1/2}\left(z\right)$ are the modified spherical Bessel functions of the first kind, and $Y_{k,m}\left(\theta ,\phi \right)$ are the normalized spherical harmonics. While the enumeration of spherical harmonics and thus of Steklov eigenfunctions requires the second index *m* (ranging from −k to *k*), it can be omitted in our analysis. Indeed, since $v_{0}^{\left(p\right)}=Y_{0,0}=1/\sqrt{4\pi R^{2}}$ is a constant, the integral of $v_{k}^{\left(p\right)}$ over Γ vanishes for all k>0 due to the orthogonality of spherical harmonics. As a consequence, one has

(61) $$c_{k}^{\left(p\right)}\left(x_{0}\right)=\frac{i_{0}\left(r_{0}\sqrt{p/D}\right)}{i_{0}\left(R\sqrt{p/D}\right)}\delta_{k,0},$$

where $r_{0}=\left|x_{0}\right|$ is the radial coordinate of the starting point. We then obtain an explicit solution in the Laplace domain:

(62a) $$\begin{array}{cc} {\tilde{Q}}_{0}\left(p|r_{0}\right) & =\frac{1}{p}-\frac{i_{0}\left(r_{0}\sqrt{p/D}\right)}{p\;i_{0}\left(R\sqrt{p/D}\right)}\Upsilon \left(\mu_{0}^{\left(p\right)}\right), \end{array}$$

(62b) $$\begin{array}{cc} {\tilde{Q}}_{n}\left(p|r_{0}\right) & =\frac{i_{0}\left(r_{0}\sqrt{p/D}\right)}{p\;i_{0}\left(R\sqrt{p/D}\right)}{\left[\Upsilon \left(\mu_{0}^{\left(p\right)}\right)\right]}^{n}\left(1-\Upsilon \left(\mu_{0}^{\left(p\right)}\right)\right), \end{array}$$

with $i_{0}\left(z\right)=\sinh\left(z\right)/z$. In turn, the Laplace inversion needs to be performed numerically for a given moment-generating function Υ(μ).

**Figure 4:**
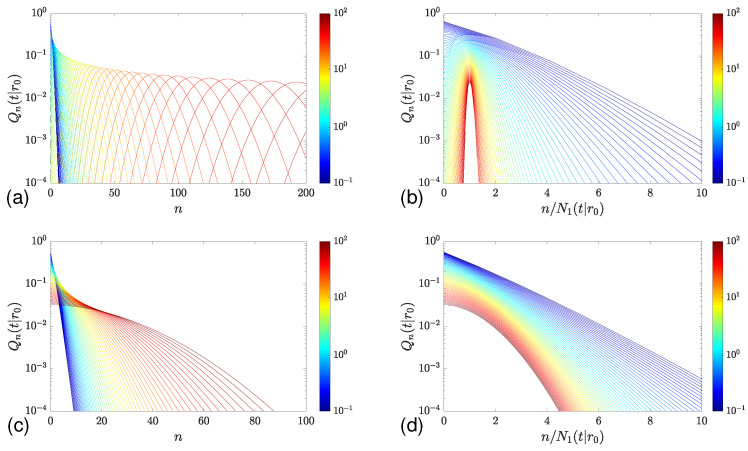
Statistics of adsorption events on the adsorbing sphere of radius R=1 with a constant reactivity q=1 (top panels) and with the Mittag–Leffler adsorption model with α=0.5 and $\ell_{0}=1$ (bottom panels). (**a**,**c**) $Q_{n}\left(t|R\right)$ as a function of *n* for 64 values of *t*, chosen equidistantly on logarithmic scale, from $t=10^{-1}$ (dark blue) to $t=10^{2}$ (dark red), with D=1. (**b**,**d**) The same probabilities with *n* rescaled by the mean number of adsorptions.

Figure 4a illustrates the statistics of adsorption events on the unit sphere with a constant reactivity *q*. A visual comparison of this figure with Figure 2a highlights significant differences. While the dependence of $Q_{n}\left(t|x_{0}\right)$ on *n* remains almost flat for diffusion in the half-line, there is a distinct maximum of $Q_{n}\left(t|r_{0}\right)$ for each *t* large enough. In other words, at long times, it is highly unlikely to experience a small number of adsorptions in the case of molecular diffusion inside a bounded domain. The maximum of $Q_{n}\left(t|r_{0}\right)$ is shifted to larger values of *n* as time increases. Moreover, the distribution becomes more and more concentrated around the mean value, as discussed in Section 2.2.1. To outline this narrowing effect, Figure 4b presents the same distribution but plotted against the rescaled number $n/N_{1}\left(t|r_{0}\right)$. As time increases, this distribution becomes narrower, and its width, characterized by the coefficient of variation, vanishes.

The behavior is different for adsorption mechanisms with an infinite mean threshold $E\{\hat{\ell }\}$. Figure 4c shows the statistics of adsorptions for the Mittag–Leffler model (57) with α=0.5. This panel resembles Figure 2a for diffusion on the half-line. As shown in Section 2.2.1, the coefficient of variation approaches a constant, i.e., the distribution does not become narrower (Figure 4d).

## 5. Discussion and Conclusions

In this paper, we developed a general theoretical framework for accessing the statistics of adsorption and permeation events in molecular diffusion. These statistics depend on the geometric shape of the confinement and the adsorption/permeation mechanism on the boundary. The geometry is captured via the Steklov eigenmodes, whereas the mechanism is introduced via the random threshold $\hat{\ell }$ and its moment-generating function $\Upsilon \left(\mu \right)=E\left\{e^{-\mu \hat{\ell }}\right\}$. Using the encounter-based approach, we derived the spectral expansion (17) for the Laplace transform ${\tilde{Q}}_{n}\left(p|x_{0}\right)$ of the probability $Q_{n}\left(t|x_{0}\right)$ of having *n* adsorption events up to time *t* (i.e., of $N_{t}=n$). A similar yet much more formal expansion was derived for the statistics of permeation events. On one hand, the inverse Laplace transform of ${\tilde{Q}}_{n}\left(p|x_{0}\right)$ allows one to obtain $Q_{n}\left(t|x_{0}\right)$, at least numerically. Moreover, the small-*p* asymptotic behavior of ${\tilde{Q}}_{n}\left(p|x_{0}\right)$ determines the long-time asymptotic behavior of $Q_{n}\left(t|x_{0}\right)$, as we illustrated for bounded and unbounded domains. In fact, $Q_{n}\left(t|x_{0}\right)$ strongly depends on the chosen adsorption mechanism via the asymptotic expansion of the moment-generating function Υ(μ), in particular, whether the mean threshold $E\{\hat{\ell }\}$ is finite or not. On the other hand, if the molecule has an exponentially distributed random lifetime, the Laplace transform ${\tilde{Q}}_{n}\left(p|x_{0}\right)$ directly provides the statistics of adsorption events realized prior to degradation, passivation, or irreversible transformation of the molecule.

We also discussed the behavior of the mean number of adsorptions $N_{1}\left(t|x_{0}\right)$, its second moment $N_{2}\left(t|x_{0}\right)$, and the resulting standard deviation. Moreover, we introduced the effective reactivity $\kappa (t|x_{0})$ as the ratio between the mean number of adsorptions and the mean boundary local time $L(t|x_{0})$. The latter can be understood as a proxy for the mean number of adsorption attempts, whereas $N_{1}\left(t|x_{0}\right)$ is the mean number of their successful outcomes. Expectedly, $\kappa (t|x_{0})$ is shown to be constant in the common setting of a constant reactivity, which is characterized by the exponential distribution of the threshold $\hat{\ell }$ or, equivalently, by the Robin boundary condition. In general, however, the effective reactivity is not constant, and its time and space dependence reveals the impact of the adsorption mechanism. In particular, at long times, $\kappa (t|x_{0})$ can either approach a constant or vanish, depending on the adsorption mechanism. This behavior is rather universal for bounded confining domains. In turn, the short-time behavior is more sensitive to the particular choice of the moment-generating function Υ(μ) and may even depend on the starting point $x_{0}$.

The general theoretical framework was illustrated on two practically relevant examples of a flat surface and a spherical surface. In the former case, the Steklov spectrum contains a single, fully explicit eigenmode that dramatically simplifies the analysis. In particular, we managed to obtain $Q_{n}\left(t|0\right)$ explicitly for two adsorption mechanisms: a constant reactivity and a fixed threshold. For a spherical boundary, the spectrum of the Steklov problem contains infinitely many eigenmodes but the rotational symmetry implies that only the principal eigenmode contributes to the spectral expansion (17). While the Steklov–Neumann spectral problem and the consequent spectral expansions are applicable for general confining domains with Lipschitz boundaries (e.g., polygons), one needs to resort to numerical tools for computing the spectrum (see [92] and references therein). Further investigation of the statistics of adsorptions on irregular boundaries presents an interesting perspective of this work.

The progress in optical imaging allows one to gather significant amounts of single-particle trajectories in various biological and soft matter systems. The statistics of adsorption and permeation events can thus potentially be obtained from these data. One can then formulate a number of optimization and inverse problems. For instance, what can one say about the geometric confinement and/or the adsorption/permeation mechanism from the observed statistics? Is it possible to optimize the shape of the substrate to increase or decrease the mean number of adsorption events or to reshape their distribution? If such optimal solutions exist, how do they match with the actual structure of biological and chemical systems? The developed formalism provides a mathematical foundation for addressing these challenging problems in the future.

## Data Availability

Data are contained within the article.
